# Unprovoked Spontaneous Kidney Rupture (Wunderlich’s Syndrome) Managed by Renal Artery Embolization

**DOI:** 10.7759/cureus.47367

**Published:** 2023-10-20

**Authors:** Abdulmalek Alzahrani, Mohammed Alsayed, Rana Alkhaibari, Ahmad T Alharbi, Badr Bannan, Zergham Zia

**Affiliations:** 1 Radiology, King Faisal Specialist Hospital and Research Centre, Jeddah, SAU; 2 Diagnostic Radiology, King Faisal Specialist Hospital and Research Centre, Jeddah, SAU

**Keywords:** flank pain, active bleeding, renal artery embolization, spontaneous kidney rupture, wunderlich's syndrome

## Abstract

Wunderlich's syndrome is a rare, unfamiliar disease that can present with flank pain, flank mass, and hypovolemic shock without any history of trauma. In this article, we present a sudden, unprovoked kidney rupture managed by renal artery embolization. This report emphasizes the importance of early referral and prompt management, which can be lifesaving.

## Introduction

Spontaneous kidney rupture (SKR) is a rare, unfamiliar presentation to the emergency department and can be lethal [1-4]. The clinical presentation can vary and be non-specific [1]. Thus, an actual understanding of the disease process can aid in decision-making and improve patient outcomes [1,2]. Clinical awareness and appropriate management can be lifesaving [1,5]. Here, we present a case of unprovoked spontaneous kidney rupture managed by renal artery embolization. We report our experience with transcatheter arterial embolization (TAE) using different embolic materials to emphasize how the interventional radiology team can treat a potentially lethal condition if recognized early and referred appropriately.

## Case presentation

**Figure 1:**
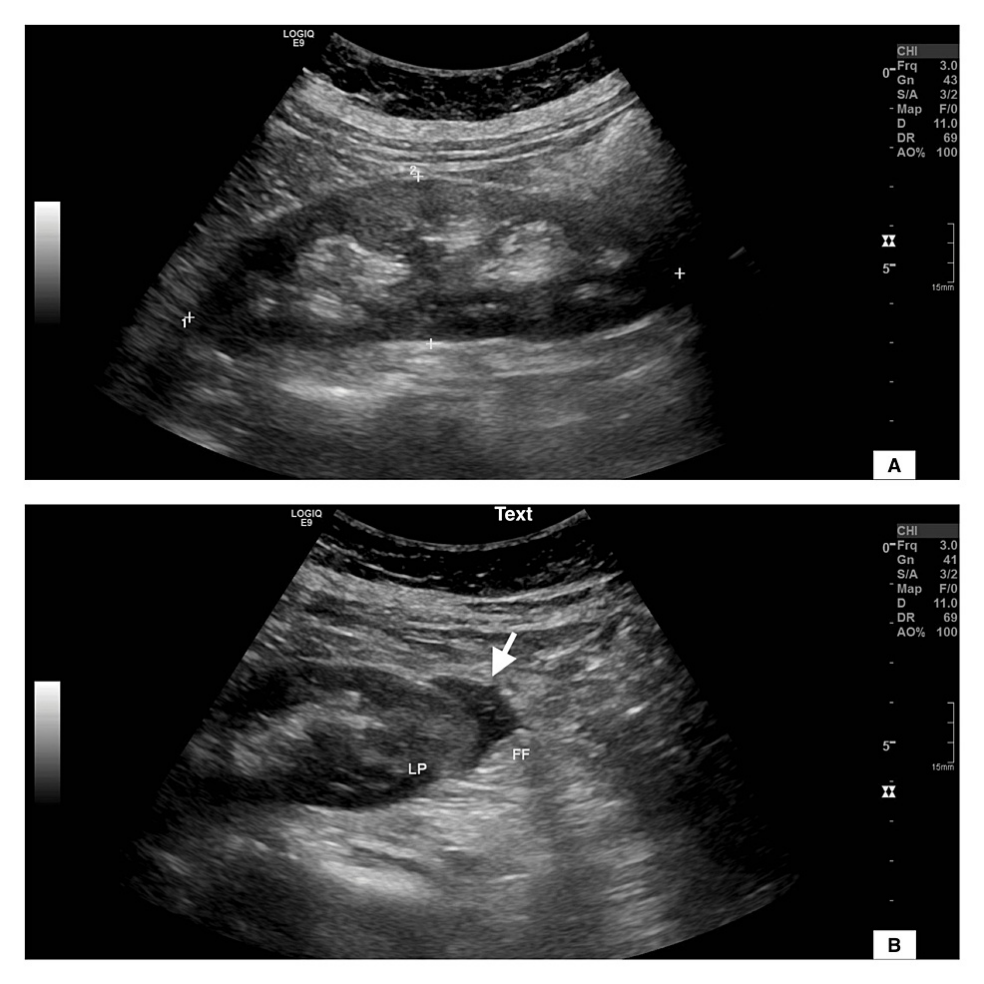
Greyscale ultrasound images in the longitudinal plane Seen is an unremarkable right kidney (A) apart from the minimal perinephric free fluid (white arrow in image B).

A 62-year-old female presented to the emergency department complaining of high-grade fever, dark urine, pleuritic chest pain, and right flank pain. She had a history of diabetes mellitus type 2, chronic thromboembolic pulmonary hypertension (CTEPH) on lifelong rivaroxaban with an inferior vena cava (IVC) filter, resected papillary thyroid carcinoma, and resected meningioma. Lab results revealed an elevated white blood cell count, high creatinine, and positive urinary ketones. A ventilation perfusion scintigraphy was done to investigate pleuritic chest pain. It demonstrated a mismatch defect suggestive of acute vs. chronic pulmonary embolism. Hence, a therapeutic dose of apixaban was initiated upon discontinuation of rivaroxaban. The patient was admitted for management of diabetic ketoacidosis (DKA) due to presumed pyelonephritis. As a result, the DKA protocol with antibiotic coverage was initiated. Further evaluation of the right flank pain by ultrasonography demonstrated minimal perinephric free fluid and an otherwise unremarkable right kidney (Figure 1).

**Figure 2:**
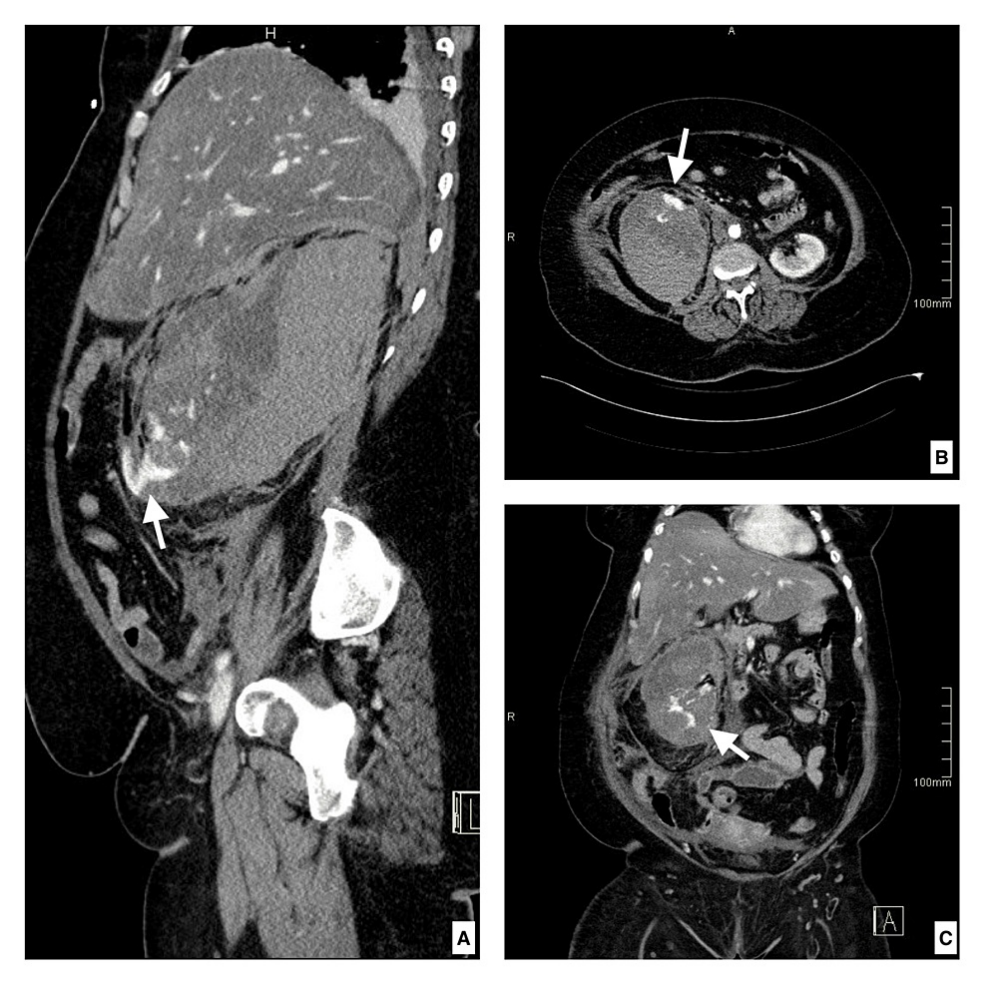
Enhanced sagittal, axial, and coronal CT scans acquired in the arterial phase Seen is a shattered kidney with cortical distortion and active contrast extravasation (white arrows in images A, B, and C).

Three days later, the patient developed severe progressive right flank pain, dizziness, fatigue, and hypovolemic shock with a significant hemoglobin drop from 14 to 8 g/dL. Due to the patient’s known history of anticoagulant treatment, intra-abdominal bleeding was suspected. Therefore, enoxaparin was discontinued, and an urgent abdominal CT scan with contrast was performed, demonstrating a shattered right kidney with significant cortical distortion, retroperitoneal hemorrhage, and active contrast extravasation in keeping with active bleeding (Figure 2).

**Figure 3:**
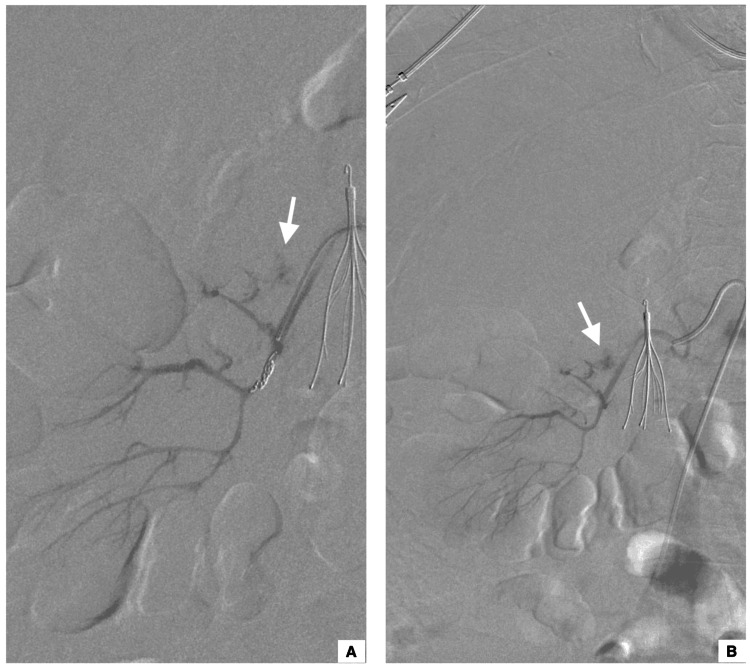
Digital subtraction renal angiography images showing an active contrast extravasation from the right renal artery (white arrows in images A and B)

The patient was given two units of packed red blood cells and shifted immediately to the angiography suite. The right common femoral artery was accessed using a Simmons (SIM1) catheter, and the right renal artery was selected. A selective angiogram of the right renal artery demonstrated active contrast extravasation (Figure 3).

**Figure 4:**
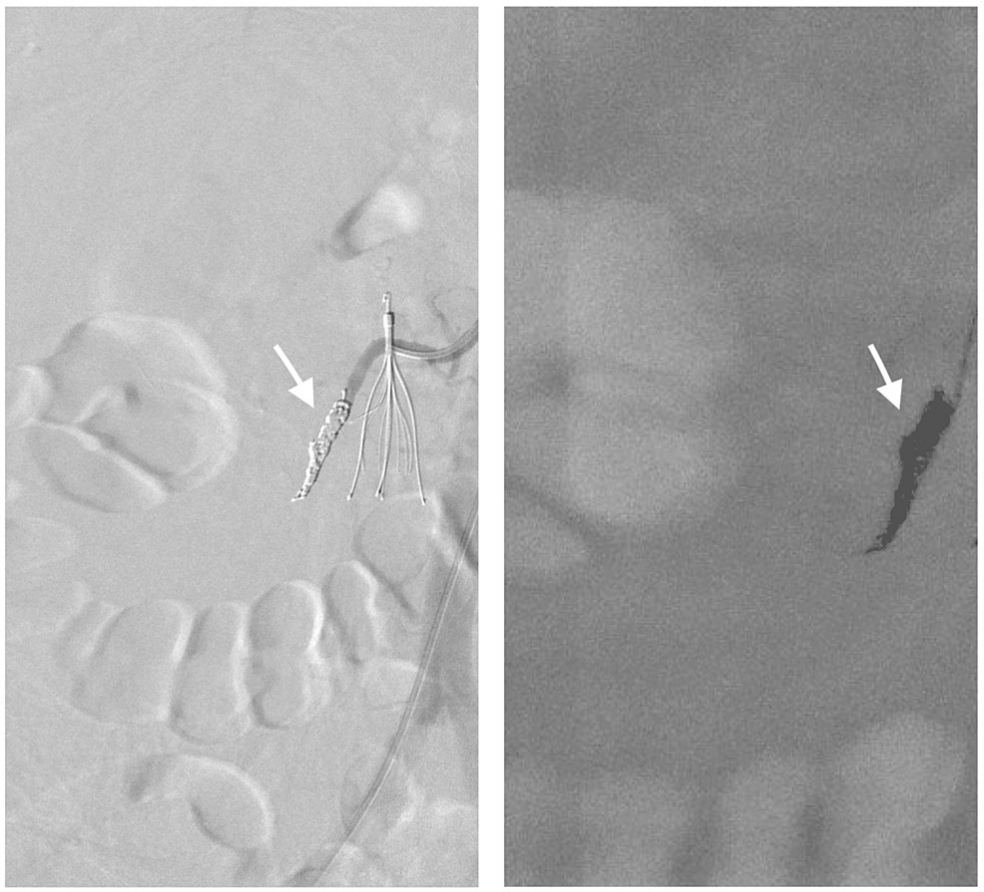
Digital subtraction renal angiography images showing the successful coil embolization of the right renal artery (white arrows in both images)

A microcatheter was advanced distally in the renal artery, and then embolization was performed using multiple 0.018-inch hydrogel-coated pushable coils of different sizes, including 1x (2 mm x 2 cm), 1x (4 mm x 4 cm), 5x (4 mm x 6 cm), and 3x (6 mm x 6 cm) Azur coils (AZUR 18; Terumo Medical Corp., Tokyo, Japan). A selective right renal artery angiogram revealed successful embolization with no further contrast extravasation (Figure 4).

**Figure 5:**
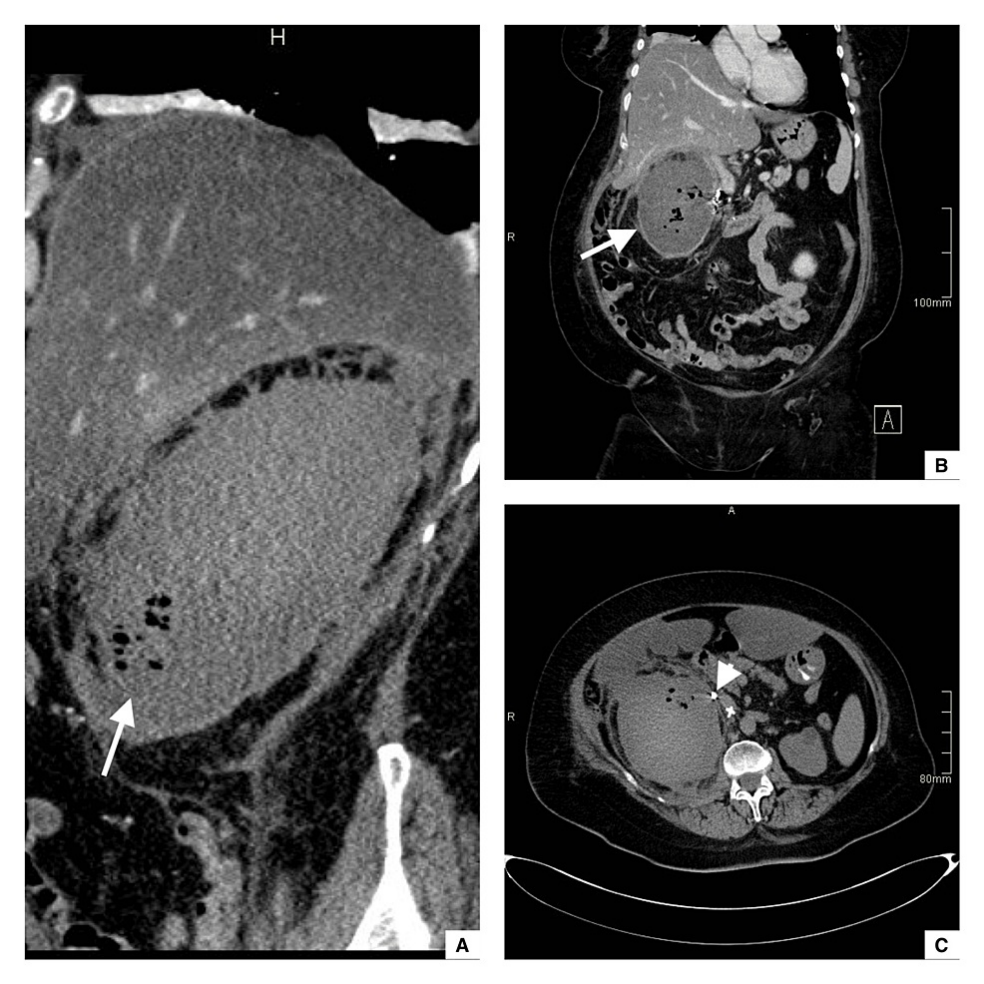
Enhanced sagittal and coronal CT scan A and B: An infected hematoma with air pockets (white arrows), C: Coil embolization (arrowhead)

Four days later, enoxaparin was resumed. Approximately eight weeks after embolization, the patient was admitted to the hospital after repeated visits to the emergency department due to high-grade fever and right flank pain associated with leukocytosis and high C-reactive protein despite antibiotic treatment. A CT abdomen was performed, revealing nephric collection with air pockets suggestive of an infection (Figure 5).

The patient underwent an elective total nephrectomy. The resected specimen was sent for investigation of the underlying pathology, which revealed coagulative necrosis, hemorrhage, and fibrin consistent with prior embolization. No malignant tissue was identified.

## Discussion

This rare entity was first described in the literature review as Wunderlich's syndrome in 1856, characterized by Lenk's triad: acute flank pain, flank mass, and hypovolemic shock [1,2]. Angiomyolipoma (AML), followed by renal cell carcinoma (RCC), is considered the most common benign and malignant neoplasms, which account for 57% to 73% of the causes of this syndrome. However, other less common causes such as vascular abnormalities, hereditary and acquired renal cystic diseases, renal infections, calculus disease, anticoagulant therapy, and coagulation disorders have been associated [2,3,6]. Computed tomography evaluation is a cornerstone investigation that reveals the underlying pathology and provides a roadmap for management [4].

Early referral to interventional radiology can facilitate diagnosis, confirmation, and treatment, as catheter angiography helps in identifying the bleeding artery and blocking the source with embolization [4]. Selective embolization has been successfully performed in the past for patients with various conditions affecting the kidneys. For example, Pekka et al. reported that two patients known to have tuberous sclerosis presented with spontaneous rupture of the kidney and were managed by selective angiography embolization [7]. Another patient presented with a severe bleeding episode from a ruptured malignant oncocytoma and benefited from therapeutic angiography before undergoing invasive nephrectomy [7]. In these cases, the bleeding kidney was embolized using ethanol, which effectively stopped the bleeding and allowed for more time to stabilize the patient's general condition before considering total nephrectomy because of the malignant oncocytoma [7]. Also, according to Grubb et al., patients benefit from successful coil embolization before total nephrectomy due to malignancy [2]. A successful transcatheter arterial embolization with the use of different embolic materials on 22 patients presented with hypovolemic shock secondary to ruptured angiomyolipoma was reported by Gong et al. [8].

In our patient, the diagnosis of spontaneous kidney rupture was made rapidly, and the patient was promptly referred to interventional radiology for catheter angiography and embolization. This procedure helped stop the acute bleeding and avoid major nephrectomy surgery for a patient who was rapidly deteriorating. Unfortunately, due to the persistent complications of uncontrolled infection and the development of renal abscesses despite antibiotic coverage, the patient eventually required a total nephrectomy. However, this was done when the patient was stable and on an elective basis.

Our experience with this rare entity adds to the limited literature on spontaneous kidney rupture and suggests that catheter embolization should be considered the first treatment of choice in acute settings. Although nephrectomy has been provided safely as the first-line treatment in the literature, it does carry the risk of mortality [9]. Bensalah et al. reported four patients who were on anticoagulants due to varied medical conditions, presented with spontaneous kidney rupture, and underwent total nephrectomy, which resulted in three tragic deaths in the immediate postoperative period [6]. On the other hand, managing patients on anticoagulants with renal artery embolization had better outcomes, rapid recovery, shorter hospital stays, and lower complication rates [6,9]. 

## Conclusions

To summarize, we report this rare case of unprovoked spontaneous kidney rupture, demonstrating a serious life-threatening condition that must be rapidly differentiated from other abdominal emergencies. As in our vignette, early clinical suspicion, appropriate investigation by CT scan, and immediate cessation of blood thinner are essential. With the advancement in imaging technology and minimally invasive procedures, the prompt involvement of interventional radiology plays a crucial role in safe and effective management with embolization, which may improve patient outcomes and avoid unnecessary major surgical interventions.
